# Does Inflammatory Prognostic Index Predict Postoperative Outcomes in Coronary Artery Bypass Grafting?

**DOI:** 10.3390/jcm15124547

**Published:** 2026-06-11

**Authors:** Mustafa Karaarslan, Osman Fehmi Beyazal, Zeki Temizturk, Bedirhan Cevik, Nihan Kayalar, Mehmed Yanartas

**Affiliations:** 1 Department of Cardiovascular Surgery, Basaksehir Cam and Sakura City Hospital, Istanbul 34480, Turkey; karaarslanmustafa123@gmail.com (M.K.); zekitmztrk5806@gmail.com (Z.T.); nkayalar@hotmail.com (N.K.); myanartas@yahoo.com (M.Y.); 2 School of Medicine, Istanbul Medipol University, Istanbul 34810, Turkey; bedirhancevik@gmail.com

**Keywords:** inflammatory prognostic index, inflammation, prognosis, coronary artery bypass grafting, mortality

## Abstract

**Background**: The inflammatory prognostic index (IPI) is a novel hematological parameter that reflects both inflammatory burden and immune status. Despite several studies on this new index, the prognostic value of IPI in CABG remains unclear so far. This study aimed to evaluate whether the IPI could serve as a predictor of postoperative outcomes in patients undergoing coronary artery bypass grafting (CABG). **Methods**: A total of 640 patients who underwent isolated CABG between 2022 and 2025 were retrospectively analyzed. The optimal preoperative IPI cut-off value for predicting mortality was determined using receiver operating characteristic (ROC) curve analysis. The optimal IPI cut-off value was identified as 0.22 (AUC = 0.607; 95% CI: 0.468–0.747; *p* = 0.14). Based on this threshold, patients were categorized into two groups: high IPI (Group A, n = 293) and low IPI (Group B, n = 347). **Results**: Baseline demographic features, comorbid conditions, echocardiographic findings, operative variables, cardiopulmonary bypass time, cross-clamp time, and laboratory results were comparable between the groups, except for gender distribution and platelet counts. The incidence of postoperative cerebrovascular events was significantly higher in Group A. Although mortality was more frequent in Group A than in Group B (3.8% vs. 1.4%), this difference did not reach statistical significance (*p* = 0.06). Additionally, patients in Group A had significantly longer intensive care unit (ICU) stays (mean: 3 vs. 2.6 days, *p* = 0.01) and overall hospital stays (9.5 vs. 8.4 days, *p* = 0.002). Multivariate regression analysis demonstrated that gender, diabetes mellitus, hypertension, and IPI were not independently associated with mortality. **Conclusions**: The IPI was associated with longer ICU and hospital stays in isolated CABG patients. These results support the use of IPI as a potential prognostic index for CABG patients.

## 1. Introduction

Coronary artery bypass grafting (CABG) remains the most frequently performed procedure in cardiac surgery. Following isolated CABG, new-onset atrial fibrillation (AF) occurs in 30% of cases, prolonged ventilation in 12.3%, renal failure in 4.5%, reoperation in 3.5%, stroke in 1.9%, and sternal wound infection in approximately 0.4% [1]. Various scoring systems, such as the European System for Cardiac Operative Risk Evaluation (EuroSCORE) II and the Society of Thoracic Surgeons (STS), have been developed to predict these complications, identify high-risk patients, and implement necessary precautions. More recently, simpler and more readily applicable biomarkers, such as the inflammatory prognostic index (IPI), have been introduced as potential alternatives for risk assessment [2].

IPI is a novel hematological parameter that reflects both inflammatory burden and immune status. IPI was first defined by Dirican et al. as a prognostic index, which is calculated using the formula IPI = C-reactive protein × neutrophil/lymphocyte ratio/albumin in patients with non-small cell lung cancer [3]. Additionally, a high IPI has been found to independently predict mortality in type A aortic dissection [4]. Another study showed that IPI can predict late-term mortality in aortic valve replacement procedures [5]. It has also been suggested that IPI may serve as a useful marker for predicting new-onset atrial fibrillation after CABG [2]. Despite several studies on this new index, none have comprehensively compared all postoperative outcomes with IPI in CABG patients. Accordingly, this study aimed to investigate whether preoperative IPI can serve as a predictor of postoperative outcomes in patients undergoing CABG.

## 2. Methods

This retrospective, single-center observational study comprised 640 consecutive patients. All individuals aged ≥18 years who underwent isolated coronary artery bypass grafting (CABG) at Istanbul Basaksehir Cam and Sakura City Hospital between 1 January 2022 and 5 November 2025 were included. Patients undergoing cardiac procedures other than CABG, those who had concomitant cardiac procedures in addition to CABG, those who had off-pump procedures without cardiopulmonary bypass (CPB), reoperative cases, and patients requiring perioperative intra-aortic balloon pump (IABP) or extracorporeal membrane oxygenation (ECMO) support were excluded. Additionally, patients with chronic renal failure (CRF), a history of malignancy, prior organ transplantation, or rheumatologic disease were excluded. The optimal preoperative inflammatory prognostic index (IPI) threshold for predicting mortality was determined using receiver operating characteristic (ROC) curve analysis. Based on this cut-off value, the study population was stratified into two groups: Group A (n = 293), consisting of patients with IPI values above the threshold, and Group B (n = 347), comprising those with values below the threshold.

Comprehensive data were collected for all patients, including demographic characteristics, comorbid conditions, transthoracic echocardiographic (TTE) findings, and detailed laboratory parameters. In addition, perioperative variables such as vasoactive inotropic score (VIS), CPB time, and aortic cross-clamp (XCL) time were recorded. Postoperative parameters included drainage volume and a wide range of complications, including postoperative atrial fibrillation (POAF), cerebrovascular accident (CVA), re-exploration, continuous renal replacement therapy (CRRT), sternal wound infection, percutaneous coronary intervention (PCI), gastrointestinal bleeding, tracheostomy, thoracentesis, duration of mechanical ventilation, length of intensive care unit (ICU) stay, total hospital stay, and in-hospital mortality. A CVA was clinically defined as any focal neurological deficit manifesting during the postoperative follow-up period, objectively validated via neuroimaging modalities, and officially corroborated by expert neurological consultation.

The study protocol was approved by the Istanbul Basaksehir Cam and Sakura City Hospital Ethics Committee (Decision No.: 2025-421, dated 8 December 2025) and was conducted in accordance with the principles of the Declaration of Helsinki. Given the retrospective design of the study, informed consent was not required. No artificial intelligence-assisted technologies were used in the preparation of this manuscript.

### Statistics

All statistical analyses were performed using SPSS software (version 27.0; IBM Corp., Armonk, NY, USA). Continuous variables were summarized as minimum, maximum, median, and interquartile range (IQR), whereas categorical variables were expressed as frequencies and percentages. The distribution of continuous variables was evaluated using the Kolmogorov–Smirnov test. Depending on the distribution characteristics, comparisons between groups were conducted using the independent samples *t*-test for normally distributed variables or the Mann–Whitney U test for non-normally distributed variables. Categorical variables were compared using the Pearson chi-square (χ^2^) test or Fisher’s exact test, as appropriate. ROC curve analysis was employed to determine the optimal cut-off value of the IPI for predicting mortality. To identify independent determinants of mortality rate, multivariate logistic regression analysis was performed, including the most common comorbid diseases, gender (where differences were found between groups), and IPI. A *p*-value of <0.05 was considered statistically significant.

## 3. Results

**Figure 1:**
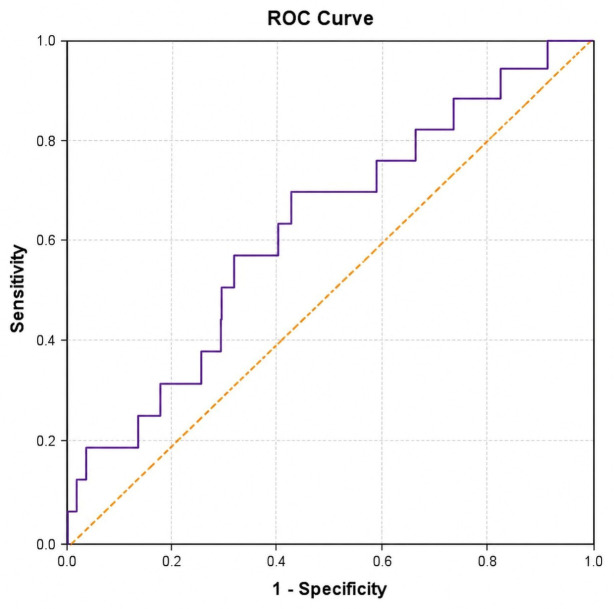
Result of ROC analysis for the inflammatory prognostic index.

Patient demographics, comorbid conditions, medication profiles, TTE findings, and operative characteristics are summarized in Table 1. The mean age was 60 ± 8.8 years, and 524 (81.9%) of the patients were male. The mean follow-up period was 420.9 ± 262 days. ROC analysis was performed with IPI to evaluate mortality prediction (Figure 1). The cut-off value for IPI was found to be 0.22 (AUC = 0.607 [95% CI: 0.468–0.747], *p* = 0.14) (sensitivity = 0.688, specificity = 0.558). The patients were divided into two groups based on whether their IPI was above or below this value. Female gender was more common in Group A than in Group B (68 (23.2%) vs. 48 (13.8%), respectively, *p* = 0.002).

No significant differences were observed between the groups with respect to baseline demographic characteristics or comorbid conditions, except for gender. Preoperative left ventricular ejection fraction (EF) and tricuspid annular plane systolic excursion (TAPSE) were comparable between the groups (median: 55% vs. 55%, *p* = 0.07; and 23 mm vs. 23 mm, *p* = 0.21, respectively). Likewise, postoperative EF and TAPSE values did not differ significantly between the groups. Postoperative VIS was also similar in both groups. In addition, there were no significant differences in operative characteristics, including rates of emergency surgery, minimally invasive procedures, coronary endarterectomy, and use of the left internal thoracic artery. The number of bypass grafts was comparable between the groups (median: 3 vs. 3, *p* = 0.78). Furthermore, CPB duration and XCL time were not significantly different between the groups (median: 131 vs. 127 min, *p* = 0.14; and 80 vs. 79 min, *p* = 0.19, respectively). Postoperative bleeding volumes were also similar (median: 700 vs. 750 mL, *p* = 0.48).

Laboratory parameters are summarized in Table 2. Preoperative white blood cell (WBC) counts were significantly higher in Group A compared to Group B (*p* < 0.001). In parallel, neutrophil counts were elevated, whereas lymphocyte counts were reduced in Group A (*p* < 0.001 for both), resulting in a significantly higher neutrophil-to-lymphocyte ratio (NLR) in this group (*p* < 0.001). No statistically significant differences were observed between the groups with respect to preoperative hematocrit, creatinine, sodium, potassium, alanine aminotransferase, aspartate aminotransferase, or HbA1c levels. Platelet counts were modestly but significantly higher in Group A than in Group B (*p* = 0.02). In addition, C-reactive protein (CRP) levels were significantly elevated, while albumin levels were significantly lower in Group A (*p* < 0.001 for both). As expected, IPI values were markedly higher in Group A compared to Group B (median: 0.62 vs. 0.07, respectively; *p* < 0.001). Postoperatively, WBC, platelet, and CRP levels remained significantly higher in Group A (*p* < 0.001). No significant differences were identified between the groups in other postoperative laboratory parameters.

Postoperative outcomes are summarized in Table 3. No statistically significant differences were observed between the groups with respect to postoperative exploration, requirement for CRRT, POAF, sternal wound infection, gastrointestinal bleeding, need for PCI, thoracentesis, tracheostomy, or duration of mechanical ventilation. However, the postoperative CVA rate was significantly higher in Group A than in Group B (9 (3.1%) vs. 3 (0.9%), respectively, *p* = 0.04). Mortality rates were also higher in Group A than in Group B, but the difference was not statistically significant (11 (3.8%) vs. 5 (1.4%), respectively, *p* = 0.06). In contrast, the ICU stay and hospital stay were significantly longer in Group A than in Group B (mean: 3 days vs. 2.6 days, *p* = 0.01; and 9.5 days vs. 8.4 days, *p* = 0.002, respectively).

Multivariate logistic regression analysis was performed to evaluate the independent effects of IPI and other clinical variables on mortality, with the results summarized in Table 4. No significant associations were identified between mortality and gender, diabetes mellitus, hypertension, or IPI. In contrast, advanced age (≥65 years) and the presence of chronic obstructive pulmonary disease (COPD) were found to be significant independent predictors of mortality (*p* = 0.001, OR = 8.898 [95% CI: 2.427–32.626] and *p* = 0.02, OR = 4.567 [95% CI: 1.274–16.375], respectively).

## 4. Discussion

The use of complete blood counts, basic biochemical parameters, and the scoring systems calculated from them have become increasingly common in cardiovascular diseases in recent years [6,7,8,9,10]. Due to its non-invasive, easy-to-calculate, and cost-effective nature, the IPI has garnered increasing attention from clinicians and researchers. Several studies have investigated its prognostic value, particularly in cancer patients [3,11,12]. Furthermore, the IPI has been associated with poor 90-day outcomes in patients with acute ischemic stroke [13]. Jiang et al.’s study highlights the potential for IPI to reduce mortality and readmission rates related to cardiovascular disease [14]. Karabag et al. found that the IPI independently predicted new-onset AF in patients undergoing primary PCI [15]. Oflar et al. also identified the IPI as a promising index that can aid in determining a risk estimate for major adverse cardiovascular and cerebrovascular events in patients undergoing PCI [16].

In cardiac surgery, IPI has been shown to predict mortality in aortic dissection and aortic valve replacement (AVR) [4,5]. In a study by Badem et al. on on-pump CABG patients, it was first identified as a noninvasive, easily accessible marker for predicting new-onset AF [2]. However, in that study, patients were divided into groups with and without POAF, and the groups were compared accordingly. In our study, however, we stratified the patient group according to the cut-off value for IPI and compared not only POAF and mortality but also all postoperative outcomes after CABG in detail. Thus, in this study, we compared the impact of low and high IPI values on all postoperative outcomes in more detail with a larger patient population.

Because the IPI is inherently closely related to inflammatory and immune status, we established rigorous selection criteria to independently investigate the impact of the IPI on postoperative outcomes to avoid confounding. We aimed to create a similar patient profile using strict exclusion criteria. Consequently, the included patient groups had similar basic demographic characteristics, comorbidities, TTE findings, and all operative data except for gender. In addition, there were no differences between the groups in other preoperative laboratory parameters except for platelet counts. Consequently, we found that the IPI was closely associated with CVAs, hospital stay, and ICU stay following CABG. We found more CVAs in patients with an IPI > 0.22. Similarly, we found significantly longer ICU and hospital stays in patients with an IPI > 0.22. These results support the IPI as a simple and easily calculated potential index that can be used to predict prolonged postoperative hospital stays. These results are valuable because, to the best of our knowledge, this study is the first to compare all postoperative outcomes after CABG. However, these findings still need to be confirmed by prospective studies.

In our study, there was also a trend towards a higher mortality rate in patients with an IPI > 0.22, although this was not statistically significant (*p* = 0.07). While there are few studies on this topic in cardiac surgery, IPI was found to be a predictor of mortality in AVR and CABG patients [2,5]. Another study in patients with abdominal aortic aneurysms found that IPI and multi-inflammatory index (MII) were higher in the survival group, but only MII was an independent predictor of mortality [6]. In the study by Badem et al., the IPI cut-off value for predicting mortality was 0.25 [2]. Similarly, in the study by Yazici et al. in AVR patients, the cut-off value was 0.25 [5]. In our study, we found a cut-off value of 0.22, which is close to the statistically significant threshold. However, multivariate regression analysis revealed that a high IPI did not independently predict mortality. This result in our study is currently statistically insufficient to conclude that IPI predicts mortality in CABG patients.

Another important factor to consider is the relationship between IPI and POAF. Systemic inflammation plays an important role in POAF [17]. Some studies have shown that IPI can predict AF in both PCI and CABG patients [2,15]. However, in our study, we did not find a significant difference in POAF between the groups (16.4% vs. 13.6%). This lack of significance, as seen in the mortality results, could be attributed to varying cut-off values found in different studies. For instance, Karabag et al. found a cut-off value of 17.5% in PCI patients, while Badem et al. found a cut-off value of 0.25 in CABG patients [2,15]. It is expected that cut-off values would differ widely in conditions with different pathophysiological conditions. However, we found a cut-off value for the CABG patient group similar to that of Badem et al., yet there was still no significant difference in POAF. It is widely acknowledged that POAF has multiple underlying causes, including structural, electrical, and autonomic factors [18]. Thus, IPI alone may not be sufficient for predicting POAF. Unlike previous studies, our findings suggest that IPI may not be a reliable predictor of POAF after CABG. Further research on POAF is needed to clarify this issue.

The IPI is a simple, inexpensive, and easily calculated index that has the potential to predict postoperative outcomes in cardiac surgery. By identifying and predicting high-risk patients, it can help facilitate perioperative interventions, thereby improving postoperative outcomes. We believe that future randomized controlled trials will reveal more significant findings regarding the prognostic significance of the IPI in cardiac surgery, a topic that has recently begun to be investigated.

### Limitations

The primary limitation of this study is its retrospective and single-center nature. While similar patient groups and strict selection criteria are important, the lack of a randomized controlled trial is a significant limitation. Establishing surgical risk scores, accounting for potential residual confounding, and comparing individual components of the IPI were not performed. Although we established very strict selection criteria for the groups and tried to obtain patient groups with similar characteristics, there was a difference in terms of gender. To prevent this, we specifically included gender in the multivariate analysis, but the higher prevalence of females may still affect the results. The ROC analysis was performed with mortality, and since there was no significant difference in mortality between the groups, the cut-off value found was not statistically significant. Furthermore, the association between IPI and mortality was investigated using multivariate analysis, but no significant relationship was found. The low overall rate of mortality reduces the power of this analysis. However, the difference found in terms of ICU stay and hospital stay with this cut-off value may be useful in demonstrating the prognostic potential of IPI. Nevertheless, future prospective research will shed more light on this issue.

## 5. Conclusions

In this retrospective study, the IPI was associated with longer ICU and hospital stays in isolated CABG patients. Determining the IPI score may predict whether postoperative follow-up will be prolonged. These results support the use of IPI as a potential prognostic index for CABG patients.

## Figures and Tables

**Table 1:** Comparison of patient demographics, comorbidities, medications, echocardiographic findings, and operative data between Group A and Group B.

	Group A (n = 293) IPI ≥ 0.22			Group B (n = 347) IPI < 0.22			
	Min–Max or n (%)	Median (Mean)	IQR	Min–Max or n (%)	Median (Mean)	IQR	*p*
**Demographic data**							
Gender female	68 (23.2)			48 (13.8)			**0.002**
Age (years)	36–79	61	13	34–86	60	12	0.11
Height (cm)	135–185	168	12	137–196	170	9	0.12
Weight (kg)	45–125	79	18	50–127	80	17	0.35
Body surface area (kg/m^2^)	1.26–2.42	1.89	0.21	1.44–2.42	1.91	0.21	0.15
Body mass index (m^2^)	18.7–55.6	28	5.6	18.2–42.8	28	5.6	0.72
**Comorbid diseases**							
Diabetes Mellitus	153 (52.2)			174 (50.1)			0.60
Hypertension	177 (60.4)			204 (58.8)			0.67
Chronic obstructive pulmonary disease	21 (7.2)			22 (6.3)			0.67
Cerebrovascular accident	26 (8.9)			19 (5.5)			0.09
Preoperative atrial fibrillation	2 (0.7)			3 (0.9)			0.79
Thyroid disorder	15 (5.1)			24 (6.9)			0.35
Operation with dual antiplatelet	16 (5.5)			12 (3.5)			0.21
Vasoactive inotropic score (VIS)	0–440	0 (6)	5	0–70	0 (3.4)	4	0.09
**Echocardiographic findings**							
Preop ejection fraction (%)	20–65	55	10	25–65	55	10	0.07
Postop ejection fraction (%)	20–65	55	15	25–65	55	10	0.07
Preop TAPSE (mm)	14–32	23	4	14–33	23	4	0.21
Postop TAPSE (mm)	9–29	16	5	8–27	17	4	0.28
**Operative data**							
Emergency surgery	15 (5.1)			18 (5.2)			0.96
Minimally invasive	2 (0.7)			2 (0.6)			0.86
Coronary endarterectomy	16 (5.5)			15 (4.3)			0.50
Left internal thoracic artery usage	259 (88.4)			311 (89.6)			0.62
The number of grafts	1–7	3 (3.1)	1	1–6	3 (3.1)	1	0.78
Cross-clamp time (min)	22–202	80	46	23–217	79	46	0.19
Cardiopulmonary bypass time (min)	41–407	131	55	40–287	127	52	0.14
Postop total amount of bleeding (mL)	100–2750	700	500	200–3025	750	363	0.48

IQR: interquartile range; IPI: inflammatory prognostic index; TAPSE: tricuspid annular plane systolic excursion.

**Table 2:** Comparison of laboratory parameters between Group A and Group B.

	Group A (n = 293) IPI ≥ 0.22			Group B (n = 347) IPI < 0.22			
	Min–Max or n (%)	Median (Mean)	IQR	Min–Max or n (%)	Median (Mean)	IQR	*p*
**Preop laboratory parameters**							
White blood cells (10^9^/L)	2–19.4	9.4	3.3	2.9–15.4	7.9	2.3	**<0.001**
Hematocrit (%)	20.5–51.9	41.2	6.1	25–52.5	41.4	5	0.21
Platelets (10^9^/L)	63–586	257	102	74–522	243	86	**0.02**
Neutrophil (109/L)	1.31–16.6	6	2.8	0.85–10.4	4.4	1.8	**<0.001**
Lymphocyte (109/L)	0.3–5.2	2.09	1.08	0.55–5.8	2.4	1.1	**<0.001**
NLR	0.9–30.7	2.7	1.7	0.55–8.1	1.82	1.03	**<0.001**
Creatinine (mg/dL)	0.42–2.09	0.93	0.30	0.27–1.81	0.92	0.24	0.41
Sodium (mEq/L)	124–150	139	4	127–146	139	4	0.07
Potassium (mEq/L)	2.69–5.84	4.33	0.62	3.41–5.74	4.39	0.6	0.18
Alanine aminotransferase (IU/L)	1–196	18	13	6–222	19	11	0.09
Aspartate aminotransferase (IU/L)	8–431	20	13	4–175	20	9	0.85
C-reactive protein (mg/dL)	1.3–211.2	9.3	11.8	0.2–8	1.7	1.9	**<0.001**
Albumin (g/L)	25–71	42	5	23–54	43	4	**<0.001**
HbA1c (mmol/mol)	5–13.9	6.4	2.6	4.9–16.2	6.2	2.1	0.09
Inflammatory prognostic index (IPI)	0.22–47.3	0.62	0.97	0.006–0.21	0.07	0.08	**<0.001**
**Postop 1st day laboratory parameters**							
White blood cells (10^9^/L)	5.3–50.3	17.4	8.9	3.7–44.2	15.8	6.8	**<0.001**
Hematocrit (%)	19.8–40.1	28.8	5.5	20–60	28	5.3	0.12
Platelets (10^9^/L)	65–496	202	84	53–443	178	74	**<0.001**
Creatinine (mg/dL)	0.04–3.49	1.16	0.41	0.08–4.38	1.17	0.39	0.70
Sodium (mEq/L)	133–153	143	4	135–155	142	4	0.50
Potassium (mEq/L)	2.75–5.76	4.27	0.82	2.6–5.75	4.3	0.6	0.86
Alanine aminotransferase (IU/L)	3–169	23	15	7–469	25	19	0.08
Aspartate aminotransferase (IU/L)	11–294	53	29	21–566	56	33	0.28
C-reactive protein (mg/dL)	10.3–263.3	43.6	27.2	2.1–304	37.2	21.8	**<0.001**

IQR: interquartile range; IPI: inflammatory prognostic index; NLR: neutrophil lymphocyte ratio.

**Table 3:** Comparison of postoperative outcomes between Group A and Group B.

	Group A (n = 293) IPI ≥ 0.22			Group B (n = 347) IPI < 0.22			
	Min–Max or n (%)	Median (Mean)	IQR	Min–Max or n (%)	Median (Mean)	IQR	*p*
Postoperative exploration	14 (4.8)			12 (3.5)			0.39
Cerebrovascular accident	9 (3.1)			3 (0.9)			**0.04**
Continuous renal replacement therapy	4 (1.4)			3 (0.9)			0.40
Postop atrial fibrillation	48 (16.4)			47 (13.6)			0.35
Deep sternal wound infection	14 (4.8)			8 (2.3)			0.08
Gastrointestinal bleeding	0			1 (0.3)			0.54
Percutaneous coronary intervention	1 (0.3)			2 (0.6)			0.56
Thoracentesis	19 (6.5)			23 (6.6)			0.94
Tracheostomy	2 (0.7)			1 (0.3)			0.43
Mortality	11 (3.8)			5 (1.4)			0.06
Intubation time (hours)	1–288	9	7	2–672	9	6	0.13
Intensive care unit stay (days)	1–25	2 (3)	1	1–28	2 (2.6)	1	**0.01**
Hospital stay (days)	1–104	7 (9.5)	4	2–69	6 (8.4)	3	**0.002**

IQR: interquartile range; IPI: inflammatory prognostic index.

**Table 4:** Multivariate logistic regression analysis.

	Odds Ratio	95% IC	*p*
Gender female	1.605	0.519–4.963	0.41
**Age ≥ 65**	8.898	2.427–32.626	**0.001**
Diabetes Mellitus	2.922	0.844–10.114	0.09
Hypertension	0.703	0.214–2.308	0.56
**Chronic obstructive pulmonary disease**	4.567	1.274–16.375	**0.02**
IPI	2.036	0.669–6.189	0.21

IPI: inflammatory prognostic index.

## Data Availability

This database belongs to our clinic and is not publicly accessible.

## Abbreviations

CABG	Coronary artery bypass grafting
AF	atrial fibrillation (AF)
EuroSCORE	European System for Cardiac Operative Risk Evaluation
STS	Society of Thoracic Surgeons (STS)
IPI	inflammatory prognostic index (IPI)
CPB	cardiopulmonary bypass
IABP	intra-aortic balloon pump
ECMO	extracorporeal membrane oxygenation
CRF	chronic renal failure
ROC	receiver operating characteristic
TTE	transthoracic echocardiographic
VIS	vasoactive inotropic score
XCL	cross-clamp
POAF	postoperative atrial fibrillation
CVA	cerebrovascular accident
CRRT	continuous renal replacement therapy
PCI	percutaneous coronary intervention
ICU	intensive care unit
IQR	interquartile range
EF	ejection fraction
TAPSE	tricuspid annular plane systolic excursion
WBC	white blood cell
NLR	neutrophil-to-lymphocyte ratio
CRP	C-reactive protein
COPD	chronic obstructive pulmonary disease
AVR	aortic valve replacement
MII	Multi-inflammatory index
